# Coexistence of Myelolipoma and Primary Bilateral Macronodular Adrenal Hyperplasia With GIP-Dependent Cushing's Syndrome

**DOI:** 10.3389/fendo.2019.00618

**Published:** 2019-09-11

**Authors:** Stéphanie Larose, Louis Bondaz, Livia M. Mermejo, Mathieu Latour, Odile Prosmanne, Isabelle Bourdeau, André Lacroix

**Affiliations:** ^1^Division of Endocrinology, Department of Medicine, Centre Hospitalier de L'Université de Montréal (CHUM), Montreal, QC, Canada; ^2^Department of Pathology, Centre Hospitalier de L'Université de Montréal (CHUM), Montreal, QC, Canada; ^3^Department of Radiology, Centre Hospitalier de L'Université de Montréal (CHUM), Montreal, QC, Canada

**Keywords:** BMAH, myelolipoma, Cushing's syndrome, ectopic receptor, aberrant ligands

## Abstract

**Introduction:** Adrenal myelolipomas are usually isolated benign adrenal lesions, but can be adjacent to steroid-secreting adrenocortical tumors. We studied the aberrant regulation of cortisol secretion in a 61 year-old woman with combined bilateral myelolipomas and primary bilateral macronodular adrenal hyperplasia (BMAH) causing Cushing's syndrome.

**Materials and Methods:** Cortisol response was measured during *in vivo* tests that transiently modulated the levels of ligands for potential aberrant receptors, including GIP. Response to medical therapies decreasing GIP was monitored. Expression of ACTH and of GIP receptors were examined in resected adrenal tissues by immunohistochemistry and reverse transcription polymerase chain reaction (RT-PCR).

**Results:** *In vivo*, cortisol increased in response to mixed meals (+353%), oral 75 g glucose (+71%), GIP infusion (+416%), and hLH IV (+243%). Suppression of GIP by pasireotide improved cortisol secretion but produced hyperglycemia. The left adrenal was predominantly composed of myelolipoma and strands of BMAH, while the right was mainly composed of BMAH with some foci of myelolipoma on pathology. No ACTH was detectable by immunohistochemistry in BMAH or myelolipomas tissue. Ectopic GIP receptor was confirmed by RT-PCR and immunohistochemistry in BMAH tissues but not in the myelolipomas. No germline mutations were identified in the *ARMC5* gene of the patient's leucocyte DNA.

**Conclusion:** This is the first report of interspersed myelolipoma and BMAH with GIP-dependent Cushing's syndrome. In contrast with the BMAH tissues, myelolipoma tissue did not express specific GIP receptors. The potential mechanisms responsible for the interspersed growth of those two lesions remain to be identified.

## Introduction

### Background

Primary bilateral macronodular adrenal hyperplasia (BMAH) is a rare cause of Cushing's syndrome (CS), but more frequently presents as bilateral adrenal incidentalomas with modest cortisol secretion (1). Cortisol secretion in BMAH is frequently associated with the aberrant expression and regulation by various G-protein coupled hormone receptors (GPCR). Recently, the production of ACTH was identified in clusters of steroidogenic cells in the majority of BMAH cases studied and its secretion was partially regulated by the aberrant receptors (2, 3). Myelolipomas are infrequent benign adrenal tumors composed of adipose tissue and hematopoietic elements; their pathophysiology remains unknown. They are usually asymptomatic and can be managed conservatively (4). A few cases of myelolipomas were associated with hormone hypersecretion, such as hyperaldosteronism (5, 6), pheochromocytoma (7–9), and CS (10) or with congenital adrenal hyperplasia (11). In this study, we examined the aberrant regulation of cortisol secretion in a patient with CS where BMAH and myelolipoma were irregularly mixed within each adrenal gland; cortisol secretion was regulated by GIP receptor ectopically expressed in BMAH but not in the myelolipoma tissues.

### Case Presentation

**Figure 1:**
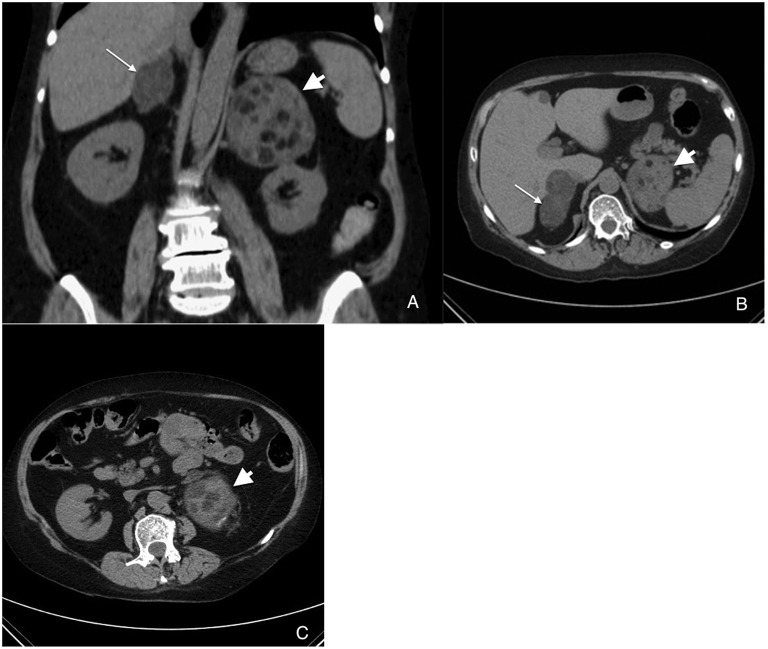
Coronal **(A)** and axial **(B,C)** views of adrenal CT scan showing bilateral adrenal enlargement with features of mixed BMAH (right; thin arrow) and myelolipoma (particularly on left; short arrow).

A 61 year-old Caucasian woman was admitted for a fibular fracture and was found to present clinical features suggestive of Cushing's syndrome (CS). Over the last 4 years, she had presented central weight gain of 12 kg, high blood pressure, osteoporosis, ecchymosis, facial hirsutism, depression, and proximal muscle weakness. Hypercortisolism was confirmed by elevated urinary free cortisol (UFC) levels (880 nmol/day, *N* < 220) and lack of serum cortisol suppression following overnight dexamethasone either at 1 mg (217 nmol/L) or 8 mg (249 nmol/L) orally. Suppressed fasting morning plasma ACTH levels basally (0.8 pmol/L, *N* = 2.0–11.0) and the absence of increase of ACTH and cortisol levels following 1 μg/kg CRH IV led to the diagnosis of ACTH-independent Cushing's syndrome. Abdominal CT and MRI studies showed bilateral enlargement of the adrenal glands (R: 6.5 × 3.5 cm, L: 8.0 × 6.9 cm) containing several nodules with heterogeneous features and density (varying from −8 to 30 HU) suggestive of mixed lesion with myelolipoma component, particularly on the left gland, while on the right hypodense regions were less present (Figures 1A–C). ^18^F-FDG PET-CT scan was not suggestive of malignancy as the maximal SUV was 2.9 in the left adrenal.

## Materials and Methods

### *In vivo* Studies

This study was approved by the ethics committee of CHUM and the patient provided written informed consent for the investigation and publication of this report. Plasma levels of cortisol, aldosterone, renin, and ACTH were measured at 30- to 60- intervals for 2–3 h during tests that transiently modulate the levels of ligands for potential aberrant receptors (12, 13). All tests were performed fasting with the patient in supine posture for at least 60 min before the tests. On day 1, an upright posture test during 2 h was followed by a standard mixed meal and by 1–24 ACTH, 250 mcg IV (Cortrosyn; Amphastar Pharmaceutical Inc, Scarborough, Ontario, Canada). On a second day, stimulation with 100 mcg GnRH IV (Factrel; Wyeth-Ayerst, Montreal, Québec, Canada) was followed 3 h later by administration of metoclopramide 10 mg orally (Sandoz, Montreal, Canada). On a third day, the administration 10 IU arginine vasopressin IM (Pitressin; Parke-Davis, Scarborough, Ontario, Canada) was performed. On a different day, an IV bolus injection of 300 IU recombinant human LH (hLH) (LHadi; Serono Laboratories, Inc., Oakville, Ontario, Canada) was performed to further evaluate a possible response in this particular case where LH levels were suppressed by exogenous chronic narcotic use and possibly by hypercortisolism. Further confirmation tests included the response to 75 g oral glucose, to a mixed meal following 100 mcg octreotide IV (Sandostatin; Novartis, Montreal, Canada), and to human glucose-dependent insulinotropic peptide (GIP; Bachem Fine Chemicals, Torrance, CA, USA) infused at a rate of 0.6 mcg/kg over 60 min, whereas the patient was receiving 150 ml/h of 10% glucose (14). A change of plasma cortisol or aldosterone levels of <25% was arbitrarily defined as no response, a 25–49% change, as a partial response, and a change of 50% or greater, as a positive response.

### Assays

Plasma cortisol, FSH, LH, TSH, and prolactin were measured by immunofluorometric assay (Bayer Immuno I System, Tarrytown, New York, USA); plasma aldosterone and renin activity were measured by RIA kits (Diagnostic Systems Laboratories, Webster, Texas, USA) and ACTH by immunoradiometric assay (Allegro, Nichols Diagnostics, San Juan Capistrano, CA, USA).

### Real-Time RT-PCR Quantification

Adrenal glands were collected from 5 patients undergoing radical nephrectomy and from this patient following each adrenalectomy and rapidly frozen in liquid nitrogen and stored at −80°C. Total mRNA was obtained from frozen tissues using TriZOL reagent (Invitrogen) and cDNA was made by iSript^TM^ cDNA Synthesis kit (BioRad). The mRNA levels of the following genes were evaluated: gastric inhibitory polypeptide receptor (GIPR), luteinizing hormone choriogonadotropin receptor (LHCGR), gonadotropin-releasing hormone receptor (GnRHR); a SYBRgreen qPCR was performed using the iQ SYBR green supermix (BioRad) on a Rotor Gene 6000 cycler as described previously (14). Results were normalized for expression of human hypoxanthine phosphoribosyltransferase 1 (hPRT) as a reference gene and were expressed relative to mRNA expression levels of a pool of normal adrenals (Clontech). Primer sequences were as follows: CCAAGCTCGGCTTTGAGAT (forward) and GTAGAGGACGCTGACCAGGA (reverse) for the GIP receptor (GIPR), CATTCAATGGGACGACACTG (forward) and GCCTCCAGGAGATTGACAAA (reverse) for the LHCG receptor (LHCGR), TGGCCTGGATCCTCAGTAGT (forward) and GAGTCTTCAGCCGTGCTCTT (reverse) for the GNRH receptor (GNRHR) and TGCTGACCTGCTGGATTACA (forward) and CCTGACCAAGGAAAGCAAAG (reverse) for the human hypoxanthine phosphoribosyltransferase 1.

### GIPR and ACTH Immunohistochemistry

Immunohistochemistry (IHC) studies were done using Benchmark ULTRA (Automated Immunohistochemistry slide staining system) from Ventana Medical System (Tucson, Arizona). A commercially available polyclonal antibody to the GIPR (1:75, LS-A3840; Lifespan Biosciences, Seattle, WA, USA) was incubated 24 min at 36°C after an EDTA treatment for 36 min in 95°C water. Detection of the staining reaction was achieved by Ultraview kit, as described previously (14, 15).

A commercially available polyclonal antibody to ACTH from Ventana Medical System (Tucson, Arizona) was incubated 32 min after treatment for 36 min in 95°C water. Detection of the staining reaction was achieved by Ultraview kit.

### Genetic Analysis for *ARMC5* Gene Mutation

After giving her written informed consent, genomic DNA was extracted from peripheral-blood leucocytes of the patient using standard protocol. *ARMC5* gene was analyzed by Next Generation Sequencing (NGS) and multiplex ligation-dependent probe amplification (MLPA) (Fulgent Genetics, Temple City, CA).

## Results

### *In vivo* Studies of Aberrant Regulation of Cortisol Secretion

**Figure 2:**
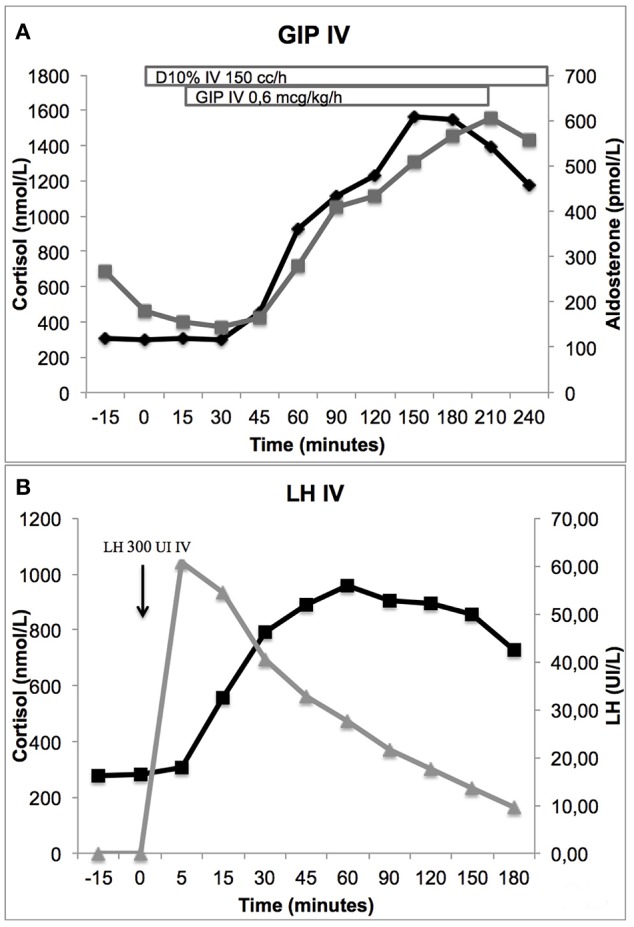
Cortisol response to stimulation by GIP 0.6 mcg/kg/h IV perfusion (416%) **(A)** and by hLH 300 IU IV (243%) **(B)**. Aldosterone response to GIP stimulation is also shown (169%) **(A)**.

Fasting plasma cortisol levels were not elevated in the morning (149–301 nmol/L), but increased during the day with peak values after meals up to 1,280 nmol/L. The *in vivo* stimulation tests showed significant cortisol increase following mixed meal (+353%), 75 g oral glucose (+71%) and GIP infusion (+416%) (Table 1 and Figure 2A), while circulating ACTH levels remained suppressed. The patient's endogenous LH levels were suppressed by regular narcotic pain therapy from fibular fracture and spinal stenosis and possibly also by hypercortisolism, explaining lack of response of LH and cortisol to LHRH administration (+2%); however cortisol levels increased following recombinant hLH injection (+243%) (Figures 2A,B). Plasma aldosterone levels also increased after mixed meal (+150%), 75 g oral glucose (+51%), GIP infusion (+169%), and hLH injection (+88%). Androstenedione levels increased following the mixed meal (+319%), GIP infusion (+410%), and hLH injection (+402%). Testosterone had a modest response to GIP (45%), as did DHEAS to the mixed meal (32%). Both had a partial response to hLH (30% and 34% respectively).

**Table 1:** Response of cortisol, aldosterone, and androstenedione plasma levels to dynamic stimulation tests modulating the levels of ligands for potential aberrant receptors.

**Tests**	**Cortisol (%)**	**Aldosterone (%)**	**Androstenedione (%)**
Posture (Supine → Upright × 2 h)	+22	+25	–
Mixed meal	+353	+151	+554
ACTH 250 mcg IV	+475	+333	–
Vasopressin 10 IU IM	−9	+13	–
Metoclopramide 10 mg PO	+49	+125	+23
GIP 0.6 mcg/kg/h IV	+416	+169	+747
Glucose 75 g PO	+71	+51	–
LHRH 100 mcg IV	+2	−1	–
hLH 300 IU IV	+243	+88	+409
Octreotide 100 mcg IV + mixed meal	+193	+7	–
Octreotide LAR 30 mg sc q 4 weeks + mixed meal	+226	+18	–
Pasireotide 900 mcg sc BID+ mixed meal	+321	+120	+280

An attempt of therapy with octreotide was initiated (first at 100 mcg sc TID, then increased to 200 mcg sc TID 3 days later, and then to Octreotide LAR 30 mg once every 4 weeks), but failed to normalize cortisol secretion. Mixed meal stimulation while on octreotide therapy produced persistent cortisol increments (+193–+853%). Administration of pasireotide 600 and 900 mcg sc BID improved cortisol levels (normal UFC and reduced post prandial elevations), but produced significant hyperglycemia. Addition of Liraglutide 1.8 mg sc to pasireotide to reduce hyperglycemia produced nausea and vomiting; the combination of fasting and pasireotide precipitated adrenal insufficiency, which required intravenous administration of hydrocortisone. Unilateral left adrenalectomy was performed, but as elevated UFC and signs of CS persisted, a right adrenalectomy was performed 1 month later. Progressive regression of CS occurred over the following 6 months and the patient is now stable on hydrocortisone and fludrocortisone replacement therapy. Screening of three first-degree relatives with dexamethasone 1 mg overnight test failed to identify any sib with abnormal cortisol secretion.

### Adrenal Pathology

**Figure 3:**
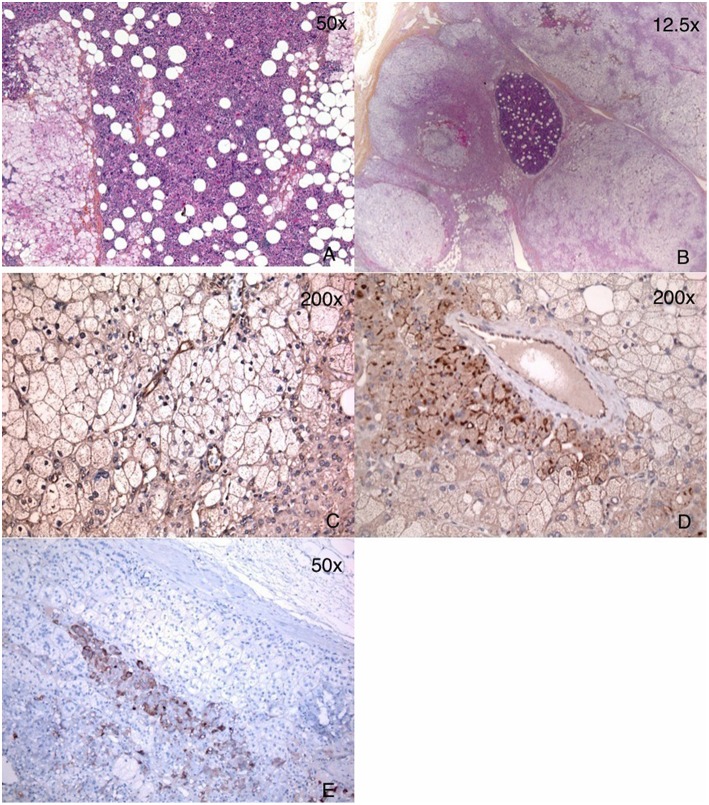
Pathology of adrenal glands showing a mixture of myelolipoma and BMAH. **(A)** Left adrenal: large areas of myelolipoma with scattered islands of adrenocorticortical cells. **(B)** Right adrenal: multiple BMAH nodules and a small area of myelolipoma. **(C)** GIPR IHC of the left adrenal gland: only endothelial staining was seen with very minimal staining in cortical adrenal cells of BMAH. **(D)** GIPR IHC of the right adrenal gland: endothelial staining and focal moderate staining in membranous pattern was seen in the cortical adrenal cells of BMAH. **(E)** ACTH IHC showed staining only in the adrenal medulla cells, and not in BMAH cells.

Pathology revealed that the left adrenal gland was predominantly composed of lipomatous tissue with areas of hematopoiesis and strands of adrenocortical nodular hyperplasia. The right adrenal was mainly composed of diffuse macronodular adrenal hyperplasia with some foci of myelolipoma (Figure 3).

### Hormone Receptor Expression by Real-Time RT-PCR

**Figure 4:**
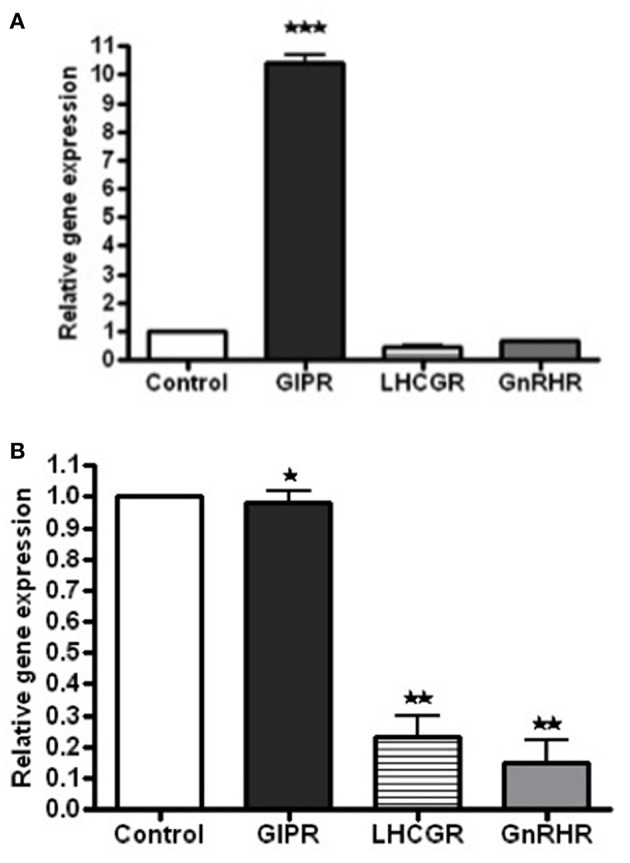
Messenger RNA (mRNA) expression levels for the receptors of GIP, LHCG, GNRH expression compared to control (pool of 5 control adrenals from Clontech) in the left and right adrenal gland tissue extracts of the patient, as determined by real-time quantitative PCR. **(A)** When compared to pool of normal adrenal glands, GIPR overexpression was found by RT-PCR in the right adrenal gland [10.5 fold higher than control adrenal tissue (***p < 0.05)]. **(B)** In the patient's BMAH/myelolipomas tissues in the left adrenal gland no increased expression of GIPR was found (*p > 0.05) and reduced expression of LHCGR and GnRHR (**p < 0.05) were found when comparing with those in the control tissues.

When compared to pool of 5 normal adrenal glands, GIPR overexpression was found by reverse transcription polymerase chain reaction (RT-PCR) in the right adrenal gland (10.5 fold higher than control adrenal tissue), but not in the left gland, which was mainly composed of myelolipoma (Figure 4). In the patient's BMAH/myelolipomas tissues, no increased expression of LHCGR nor GnRHR was found, despite *in vivo* stimulation of cortisol by LH (Figure 4).

### GIPR and ACTH Immunohistochemistry and *ARMC5* Analysis

GIP receptor overexpression was confirmed by immunohistochemistry in macronodular adrenal hyperplasia tissue, mainly in the right adrenal (Figure 3). No GIPR expression was found in the myelolipoma cells. ACTH was identified by immunohistochemistry in the patient's adrenal medulla cells, but not in the BMAH cells (Figure 3). No mutations or deletion/duplications were identified in the *ARMC5* gene of the patient's leucocyte DNA.

## Discussion

This patient presented with the first described occurrence of bilateral myelolipomas interspersed with BMAH and GIP-dependent CS. The GIP-dependent regulation of adrenal steroid secretion was suggested by stimulation of cortisol by mixed meals, oral glucose, and confirmed by direct stimulation of cortisol and aldosterone by GIP infusion. GIP receptor overexpression was confirmed by RT-PCR and immuno-histochemistry. As the combination of GIP and LH/CG receptors were previously identified in other cases of BMAH (16, 17), we also examined if recombinant LH would also regulate cortisol secretion, since endogenous LH was suppressed by opioids and possibly hypercortisolism in this patient. Recombinant LH did induce stimulation of cortisol, but as endogenous levels of LH were suppressed, it is unlikely that this aberrant receptor played a significant role in cortisol secretion. An attempt to control hypercortisolism by suppression of endogenous GIP was unsuccessful with octreotide. Pasireotide is a multi-receptor somatostatin analog, which appears via its higher affinity for sst-5 to suppress incretins and insulin more extensively than octreotide, a preferential sst-2 receptor ligand (18). In another patient with BMAH and GIP-dependent CS, octreotide and pasireotide were able to improve transiently the hypercortisolism (19); pasireotide also appeared more efficient to reduce hypercortisolism in this patient, but the important increase in glucose levels necessitated appropriate control of hyperglycemia. Based on the studies conducted on normal volunteers, the deterioration of glucose control by pasireotide is secondary to inhibitory effects on insulin and incretin secretion (18) and GLP-1 analogs and DPP-4 inhibitors appeared to better control the hyperglycemia. Unfortunately in this patient, the nausea and vomiting induced by liraglutide combined with the decreased ACTH and cortisol levels in fasting state under pasireotide led to adrenal insufficiency and thus to an indication of adrenalectomy rather than targeted medical inhibition of ligand and aberrant receptor stimulation.

In certain patients with relatively modest hypercortisolism and asymmetrical BMAH, unilateral adrenalectomy can restore normal cortisoluria, leading to long-term clinical remission, retarding hypercortisolism relapse for several years (20–23). However, this patient rapidly required bilateral adrenalectomy, possibly because BMAH tissues with ectopic GIPR were predominant in her right adrenal gland, whereas initial adrenalectomy was performed in the larger left gland, which was composed mostly of myelolipoma tissues.

The pathology of this patient confirmed the mixed and interspersed co-existence of BMAH tissues and myelolipoma tissue; this was asymmetric with predominant myelolipoma component in the left adrenal, while the right was predominantly BMAH with foci of myelolipoma.

Myelolipomas are rare benign adrenal tumors composed of mature hematopoietic tissue and fat. The absence of immature hematopoietic cells and age-related changes in composition makes them different from the bone marrow environment (24). Myelolipomas are usually asymptomatic, unless their size causes obstructive symptoms, painful hemorrhage, or rupture, which may then require surgical removal. Their prevalence is estimated to be around 6% of incidentalomas, with an increase in older age (25, 26).

A recent review by Feng et al. (27) suggests that the adipose portion of the myelolipoma derives from mesenchymal stem cells located in the adrenal blood vessels under specific stimulation. The adipocytes maturation process, which is inflammatory, causes recruitment of circulating hematopoietic progenitors to the site, which then mature using the energy produced by the adipocytes. This may create a self-growing signaling loop inside the tumor. A defective apoptosis process might also be involved in the growth of the mass (28). The paucity of molecular studies in myelolipomas has not yet allowed identifying the mechanisms of its non-malignant tumorigenesis. Clonal proliferation of both the adipose and hematopoietic tissue has been described in relation with a pattern of X-chromosome inactivation in both cell lineages (24). The clinical impact of the possible shared origin of the two cell types is still unknown.

Presence of myelolipoma has been linked to different clinical entities, such as metabolic syndrome, thalassemia, and ipsilateral adrenal tumors or functioning tissue (29, 30). It has been hypothesized that circulating or locally produced peptides or adrenal steroids may play a role in the pathogenesis of such compound tumors (31). Early data suggested a contribution of corticotropin and testosterone on the growth of such tissue in animal models (32). Previous case reports refuted this hypothesis by demonstrating the absence of androgen or ACTH specific receptors on the myelolipoma cells (33, 34), Almeida et al. recently demonstrated overexpression of androgen and MC2 receptors in giant bilateral myelolipomas from non-compliant CAH patients, in whom these tumors are frequently found bilaterally (35). The myelolipomas in that study proved to be of polyclonal origin, supporting the idea that androgen and MC2 receptor overexpression stimulated their tumorigenesis. Exogenous corticosteroid therapy is not associated with the development of myelolipoma, suggesting the absence of direct contribution of cortisol to its pathogenesis. However, glucocorticoids are known to regulate many steps in adipose tissue formation and distribution (36). It is possible that the cortisol excess contributes to fat deposition in myelolipoma, as it has been hypothesized for the formation of spinal epidural lipomatosis associated to various cortisol excess states (37, 38). The multiple endocrine hyperfunction syndromes associated with the occurrence of myelolipoma make it difficult to identify a unique stimulus to their development. Also, only case reports of myelolipomas associated with hormone secreting adrenal adenomas have been published, suggesting a specific individual predisposition to the condition, or a pathogenetic factor that has yet to be described. Finally, of the many studies on microRNA profiles in adrenal tumors (39), only one so far investigated the microRNA profile of myelolipomas (40). To our knowledge, none investigated potential common mutations between myelolipomas and BMAH.

GIP is physiologically involved in the storage of fat in adipose tissue (41). A higher GIP receptor activity has been linked to greater accumulation of fat. GIP receptor expression could therefore theoretically play a role in the pathogenesis of the accumulation of adipose tissue in the myelolipoma. However, in the present case, no GIPR overexpression was found in the myelolipoma cells, even though it was expressed ectopically in the BMAH tissue. We therefore cannot conclude on a direct effect of GIP on myelolipoma adipocytes.

New insight into the pathogenesis of CS associated with BMAH has recently been described by Louiset et al. (2). The process involves cortisol stimulation by corticotropin secreted locally by clusters of steroidogenic cells in the hyperplastic adrenals. Furthermore, aberrant ACTH secretion by BMAH cells can be regulated in part by the aberrant GPCR (including GIPR); MC2R antagonists could decrease substantially the increase in cortisol secretion provoked by GIP in BMAH tissues with ectopic GIPR. However, in the present case, no overexpression of ACTH was found in the BMAH tissue by immunohistochemistry, while it was detectable in adrenal medulla cells as found in normal adrenals (41). No overexpression of LHCGR mRNA was found despite *in vivo* response to LH, but we did not perform specific immunohistochemistry studies to examine receptor protein expression, as endogenous LH levels were not increased in this patient and was thus unlikely to contribute to steroidogenesis.

Recent studies have shown that inactivating mutations of tumor suppressor gene *ARMC5* occur in ~25% of apparently sporadic BMAH patients (42–44), and more frequently in familial clustering cases (45, 46). Inactivation of *ARMC5* does not appear to be associated with specific aberrant receptor phenotypes in BMAH as cortisol responses to upright posture, metoclopramide, and vasopressin were found in some mutated cases; however so far no patients with GIP-dependent BMAH were found to carry *ARMC5* mutation, and this was also the case in our patient (46–48). According to a recent study, GIPR over-expression in BMAH occurs from a single allele of the GIPR gene, and in certain unilateral adenoma cases resulted from gene duplication and rearrangement; however such analysis was not performed on this patient's tissues (49).

In conclusion, the close admixture of BMAH and myelolipoma tissues in this case suggests a potential physiopathologic relationship between the two entities. Since the myelolipoma tissue did not express specific GIP receptors, further research should aim to elucidate other possible stimulation mechanism of these tumors.

## Data Availability

All datasets generated for this study are included in the manuscript/supplementary files.

## Ethics Statement

The studies involving human participants were reviewed and approved by Comité d'éthique de la recherche of the Centre hospitalier de l'Université de Montréal. The patients/participants provided their written informed consent to participate in this study. Written informed consent was obtained from the individual(s) for the publication of any potentially identifiable images or data included in this article.

## Author Contributions

Study design and analysis of data were conducted by SL, LB, LM, IB, and AL. Imaging studies were performed by OP and pathology studies by ML. SL, LB, and AL wrote the manuscript which was revised and approved by all authors.

### Conflict of Interest Statement

The authors declare that the research was conducted in the absence of any commercial or financial relationships that could be construed as a potential conflict of interest.
